# Temporally consistent sequence-to-sequence translation of cataract surgeries

**DOI:** 10.1007/s11548-023-02925-y

**Published:** 2023-05-23

**Authors:** Yannik Frisch, Moritz Fuchs, Anirban Mukhopadhyay

**Affiliations:** Computer Science, Technical University Darmstadt, Fraunhoferstraße 5, 64283 Darmstadt, Hessen Germany

**Keywords:** Cataract surgery, Unsupervised image translation, Sequence translation, Temporal consistency, Generative adversarial networks, Generative models

## Abstract

****Purpose**:**

Image-to-image translation methods can address the lack of diversity in publicly available cataract surgery data. However, applying image-to-image translation to videos—which are frequently used in medical downstream applications—induces artifacts. Additional spatio-temporal constraints are needed to produce realistic translations and improve the temporal consistency of translated image sequences.

****Methods**:**

We introduce a motion-translation module that translates optical flows between domains to impose such constraints. We combine it with a shared latent space translation model to improve image quality. Evaluations are conducted regarding translated sequences’ image quality and temporal consistency, where we propose novel quantitative metrics for the latter. Finally, the downstream task of surgical phase classification is evaluated when retraining it with additional synthetic translated data.

****Results**:**

Our proposed method produces more consistent translations than state-of-the-art baselines. Moreover, it stays competitive in terms of the per-image translation quality. We further show the benefit of consistently translated cataract surgery sequences for improving the downstream task of surgical phase prediction.

****Conclusion**:**

The proposed module increases the temporal consistency of translated sequences. Furthermore, imposed temporal constraints increase the usability of translated data in downstream tasks. This allows overcoming some of the hurdles of surgical data acquisition and annotation and enables improving models’ performance by translating between existing datasets of sequential frames.

**Supplementary Information:**

The online version contains supplementary material available at 10.1007/s11548-023-02925-y.

## Introduction

**Fig. 1:**
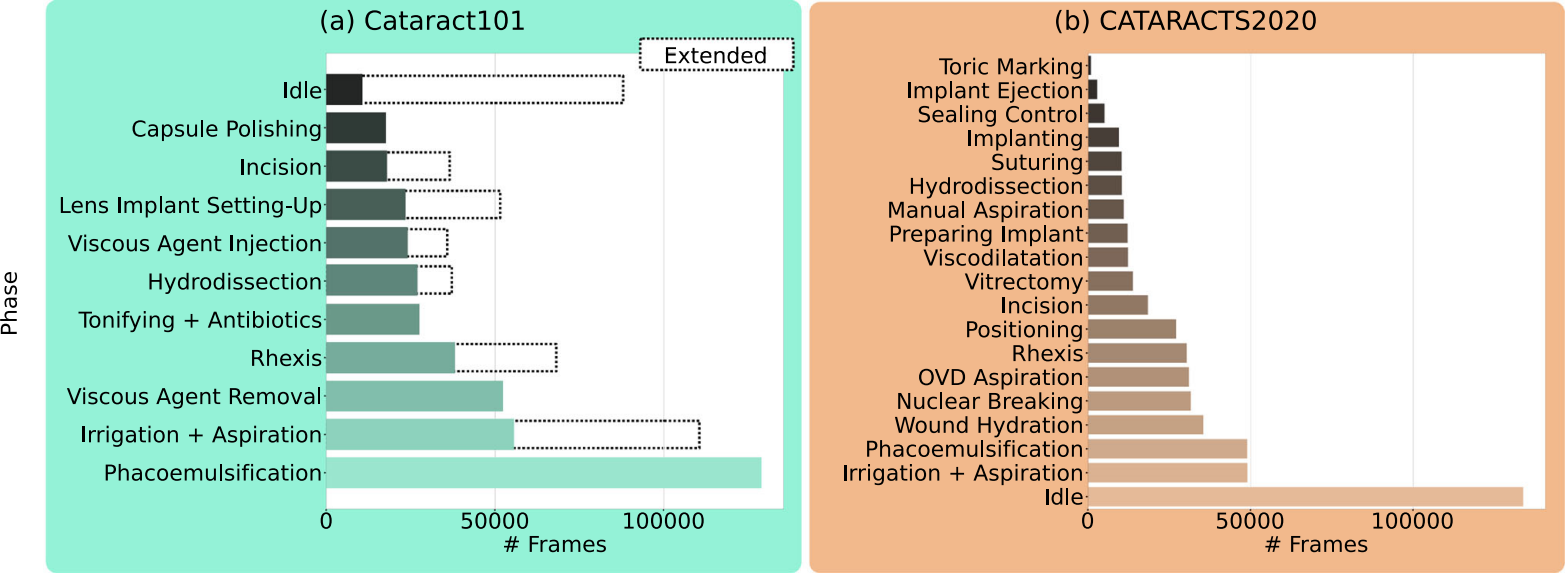
Number of available frames for training per surgical phase. Class imbalances are present, e.g., in the form of vastly less *Idle* frames for Cataract101 (**a**) compared to CATARACTS2020 (**b**). The dotted bars in **a** show the artificially increased labels for retraining the downstream task model in Chapter 4

With 4000 to 10,000 operations per million people, cataract surgeries are of high clinical relevance [1]. Nevertheless, there is an underwhelming amount of publicly available video recordings of such procedures. The two most commonly used datasets contain only 50 and 101 video sequences. In addition, they yield class imbalances, as shown in Fig. 1. Several downstream tasks for surgical data science like tool usage classification [2], surgical phase prediction [3] or anatomy and tool segmentation [4] are usually performed on single frames. Naturally, such tasks could benefit significantly from sequential image data. Imagine a surgeon classifying surgery video frames into the phases of cataract surgeries [2, 5]. Given a set of subsequent frames, this task becomes more manageable since the surgeon can make more decisive conclusions involving the displayed motion. However, gathering and annotating video sequences of cataract surgeries is costly and further complicated by privacy concerns.

**Fig. 2:**
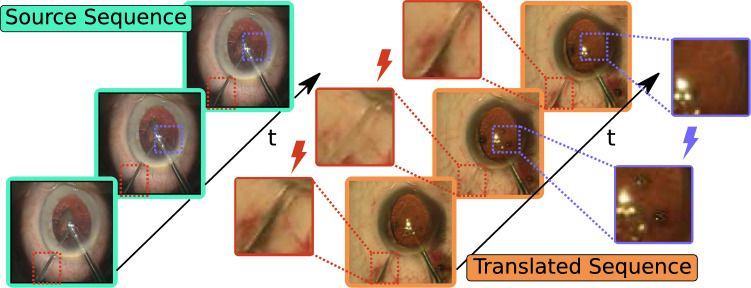
Temporal inconsistencies induced by Im2Im translation. Notice the disappearing tools (red) and the inconsistent air bubbles in the pupil (purple) when translating from the Cataract101 source domain (turquoise) to the CATARACTS target domain (orange)

Image-to-image (Im2Im) translation methods can partly navigate around these issues. They allow to translate between existing datasets to artificially extend their number of training samples and smooth out label distributions. Such translation models have shown great success in the last years in natural image domains [6, 7]. But these methods come with a big caveat when directly applied to sequence-to-sequence (Seq2Seq) translation: They are usually only trained to map between two distributions of spatial properties of images. Therefore, the translations in both directions leave much ambiguity for correctness, especially in the unpaired setting. This ambiguity results in artifacts of *temporal inconsistency*, like disappearing tools shown in Fig. 2.

Recent works in computer vision proposed spatio-temporal constraints to improve upon this issue. Such constraints are obtained either by a motion prediction model [8] or by leveraging the optical flow (OF) between samples of the input domain [9, 10]. While these models achieve increased consistency, their translation modules are relatively simple, often relying on both domains to share spatial properties and only differ in textures. When applied to spatially unaligned domains, their frame-wise translation quality usually decreases. On the other hand, disentangling content and style representations has shown success in per-frame translation methods like UNIT [7, 11, 12]. Therefore, we propose to combine the expressive power of a shared latent space with a motion translation (MT) module to impose consistency over time.

**Contributions:**

- A novel motion translation module to improve the temporal consistency of translated sequences: Our ***MotionTranslation-UNIT (MT-UNIT)*** processes both—spatial image features, as well as OF between consecutive frames—to predict motion in the target domain.
- Quantitative and qualitative evaluations of image quality and novel evaluation schemes for temporal consistency. Translating between the two most frequently used datasets of cataract surgery, we show increased performance of our method in terms of temporal consistency and quality of data for downstream tasks. Furthermore, we show that our method stays competitive in terms of the per-frame translation quality.

## Related work

Cataract surgeries have seen increased attention within the CAI community [2–5] but are relatively unexplored in terms of generative models. Existing work focuses on other modalities like fundus photographs or OCT images [13].

**Medical generative models and Im2Im translation** While not explored for cataract surgeries, generative methods have been applied to other medical domains. Such applications include synthesizing clinical data [14–16]. Often, existing data are leveraged, e.g., PET-CT translation and MR motion correction [17], translation between T1 and T2 MR images [18], simulation-to-real translation and adaptation on endovascular surgery data [19–21].

**Seq2Seq translation** To improve the temporal consistency of translated sequences, Bansal et al. [8] introduce the re-cycle constraint: They train an additional temporal prediction model and minimize the cycle loss between the predicted successive frames of the original sample and its translation. Park et al. [22] replace the temporal prediction with a warping operation using the OF obtained from the source sequence.

While these methods can achieve increased consistency, they assume spatial alignment between domains. Since this is not necessarily given, Chen et al. [10] train a module to translate the OF obtained from the source sequence into a flow field that warps the translated samples. Unlike them, we also incorporate visual information into our MT module, and also do not require an expectation–maximization-like training procedure to train it.

In related research, Rivoir et al. [20] leverage a learnable global neural texture to get view-consistent visual features from a moving camera in endovascular surgery. The method proposed by Liu et al. [9] utilizes recurrent models to incorporate temporal information, which is a promising orthogonal research direction that does not rely on predicting the optical flow between frames. Though, their method is used only to re-texture sequences, which is simpler than translating between domains of potentially spatially different features. Various methods have been proposed to solve Seq2Seq translation in a supervised way [23], which makes such translations simpler, but the extensive amount of annotations needed makes them unsuitable for our case.

**Shared latent space assumption** The incorporation of a shared latent space across domains has shown significant improvements in per-image translations [7, 11, 12]. However, it is rarely leveraged in recent work addressing temporal smoothness. To the best of our knowledge, ours is the first work extending the UNIT method [7] with a MT module for OFs. Further, this is the first application of Seq2Seq translation on the cataract surgery domain.

## Method

Let *A* and *B* be two image domains and $a:= \{a_t\}_{t=1}^T$ and $b:= \{b_t\}_{t=1}^T$ be two unpaired image sequences of equal length *T* with $a \in A$ and $b \in B$. In both directions, we seek mappings $f_A$ and $f_B$ such that the mapped sequences ${\hat{a}}:=f_A(b)$ and ${\hat{b}}:=f_B(a)$ resemble sequences from the domains *A* and *B*, respectively. Additionally, we aim for the preservation of temporal consistency based on the smooth movement assumption of natural objects.

**Fig. 3:**
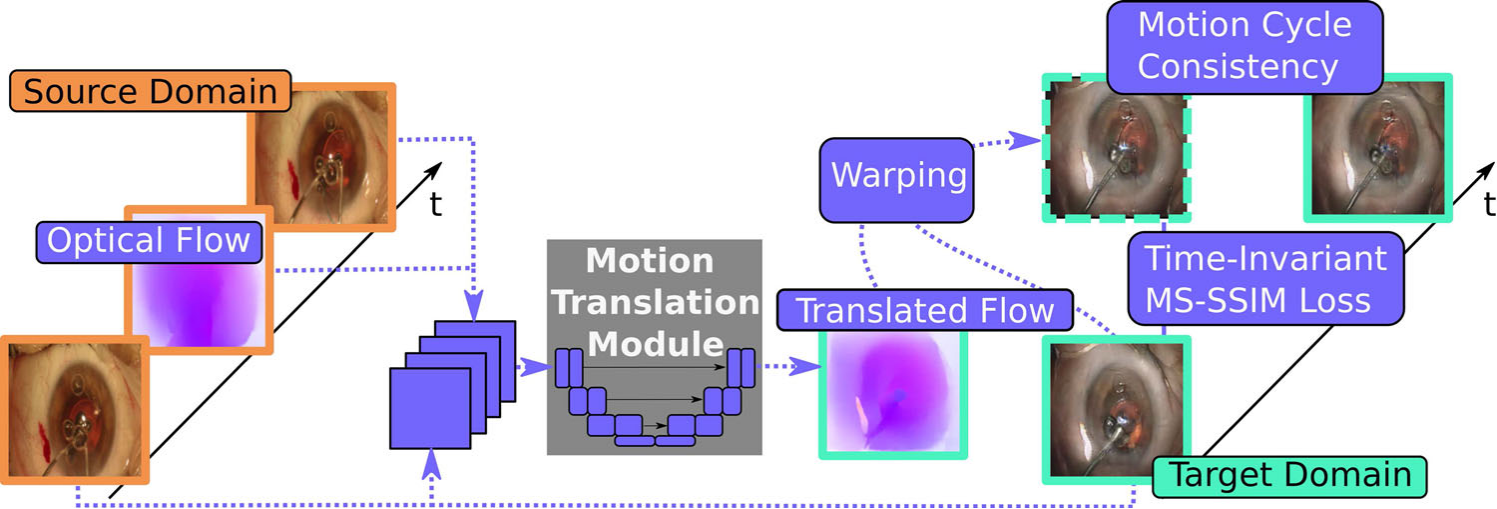
Proposed motion translation module. The OF between consecutive frames of the source domain (orange) is translated by the MT module. The module receives two frames and the OF from the source domain, concatenated with one frame of the target domain. From here it predicts flow-fields to realistically warp between translated frames of the target domain. It is trained by imposing a MS-SSIM loss between consecutive translated frames (turquoise) and a motion cycle consistency between the warped successor frame (turquoise, dashed) and the translated successor frame

We employ a variational autoencoder (VAE) structure to learn these mappings following Liu et al. [7]. The architecture consists of domain-dependent encoders $E_A$ and $E_B$ and decoders $G_A$ and $G_B$. Cross-domain mappings are then defined by $f_A(b):=G_A(E_B(b))$ and $f_B(a):=G_B(E_A(a))$. Two separate networks, $D_A$ and $D_B$, are trained to discriminate between samples originating from *A* and *B*. We add two terms to the objectives to impose temporal consistency, which are visualized in Fig. 3.

First, to learn realistic transitions of movement into the target domain, we extract the flow $F_A$ between frames $a_t$ and $a_{t+1}$ using a pre-trained *RAFT* model [24]. We then translate it as ${\hat{F}}_B = M_{AB}(a_t, a_{t+1}, sg({\hat{b}}_t), F_A)$ where $M_{AB}$ is another network trained jointly with the model’s generator part and *sg* is the stop-gradient operation. For $M_{AB}$, we deploy a UNet type architecture [25] since the multi-scale features will help the model to translate between spatially different domains. A spatio-temporal constraint similar to the re-cycle constraint [8, 10] is enforced by minimizing

1 $$\begin{aligned} {\mathcal {L}}_{\textrm{MT}_A} = \delta _\textrm{MT} ||sg({\hat{b}}_{t+1}) - {\tilde{f}}({\hat{b}}_{t}, {\hat{F}}_B)|| \end{aligned}$$

where ${\tilde{f}}$ is a warping operation using the translated flow obtained from ${\hat{F}}_B$. A short explanation of this operation is given in Chapter 2 of the supplementary material.

Second, we penalize low values of the multi-scale structural similarity index metric (MS-SSIM) between a frame of the translated sequence and its warped counterpart by

2 $$\begin{aligned} {\mathcal {L}}_{{\text {MS-SSIM}}_A} = - \delta _\textrm{SSIM} \text {MS-SSIM}({\hat{b}}_t, sg({\tilde{f}}({\hat{b}}_t, {\hat{F}}_B))) \end{aligned}$$

where $\delta _\textrm{SSIM}$ is a balancing weight. This perceptual loss penalizes significant structural changes (e.g., disappearing tools like in Fig. 2) while being more insensitive to noise than other spatial loss functions.

The equations for samples $b \in B$ are defined analogously. In Chapters 1 and 3 of the supplementary, we give details on the VAE objective and illustrate the generative pipeline. Unlike Chen et al. [10], we are including spatial and perceptual information directly into our MT. This inclusion provides the model with richer information to perform the motion translation, producing feasible motion for the target domain. Further, we do not require an EM-like training procedure since we can put a stop-gradient operation after translating the source domain samples. This allows to train the MT module together with the generators in a single optimization step. Further note that while we build on top of UNIT in our experiments, the MT module could extend any Im2Im method in theory.

## Experimental setup

In this section, we explain used data, implementation and experimental details.

**Data** The *CATARACTS2020* data [2] consist of 50 videos of cataract surgeries. 25 are used as the training set, 5 for validation and 20 for testing. The surgeries were done by three different surgeons of varying expertise level and are recorded with $1920\times 1080$ pixels and 30 FPS. Annotations for the 2020 version consist of frame-wise surgical phase annotations out of 19 phases.

The *Cataract101* dataset [5] contains 101 video recordings of cataract operations, done by four different surgeons with two levels of expertise. We split the data into 70 videos for training, 20 for validation and leave 11 for testing. The frames have a resolution of $720\times 540$ pixels, were recorded at 25 FPS and are annotated with the ten surgical phases described in the paper.

The VAE-GAN backbone relies on perceptual similarities for the discriminator to learn mappings for input images. Without any supervision or spatially similar counterparts in the other domain, the generator will not be able to learn such a mapping. Therefore we pre-filter the phases considered for the training of the domain transfer model. The pre-filtered phases are required to have clear counterparts in the other domain. These include phases 0, 3–8, 10, 13 and 14 of CATARACTS2020 and phases 0–6 and 8 of Cataract101.

**Methods and network architectures** We compare our quantitative and qualitative results for image translation quality and temporal consistency across state-of-the-art baselines. These consist of CycleGAN [6] and UNIT [7] for methods without spatio-temporal regularization. For approaches including temporal information, we chose RecycleGAN [8] next to OF-UNIT, as described in [20]. Unlike the authors in [8], we leverage a pre-trained *RAFT* model [24] instead of the Gunnar-Farneback method as a much stronger OF estimator. Our MT module consists of a U-Net [25] mapping from eleven input to two output channels. All methods were re-implemented and trained equivalently.

**Training details** Every approach was trained for 200 epochs with a batch size of 8. Images were down-sampled to a size of $192 \times 192$ pixels. The Im2Im translation models are trained on single frames, while the OF models are trained on two consecutive and RecycleGAN on three consecutive frames. We use the Adam optimizer with $\beta _1=0.5$ and $\beta _2=0.999$ and an $l_2$ weight decay of $1e^{-4}$. The learning rate was initialized with $2e^{-4}$ and decreased linearly to 0 from 100 epochs onward. All model weights were initialized using the Xavier method. We sample the original videos with a frequency of every 40th frame (0.75/0.625 FPS). The objective function weights were empirically set to $\lambda _0 = \lambda _5 = \delta _\textrm{MT} = \delta _\textrm{SSIM} = 10.0$, $\lambda _1=\lambda _3=0.1$ and $\lambda _2=\lambda _4=100.0$. All models were trained on a single Nvidia A40 GPU.

**Evaluating temporal consistency** To evaluate the temporal consistency of translated sequences, we translate random sequences of length $T=10$ out of the test splits of both datasets. We compare the differences of foreground masks *fg* obtained from consecutive frames of sequence $a \in A$ and its translation ${\hat{b}}$ by

3 $$\begin{aligned} {\mathcal {M}}^d_{TC}= & {} \frac{1}{T(T-1)} \sum _{t'=1}^T \sum _{t=1}^T d(|fg(a_t) - fg(a_{t'})|, |fg({\hat{b}}_t) \nonumber \\{} & {} - fg({\hat{b}}_{t'})|) \end{aligned}$$

where *d* is a distance function. Sequences $b \in B$ are compared to ${\hat{a}}$ analogously. Possible choices for *d* are the root mean squared error (RMSE) and the structural similarity index metric (SSIM). Little differences between the foreground masks greatly influence the RMSE, e.g., when textural details are disturbed in the translated sequence. Contrary, the SSIM only penalizes more global, structural differences. To measure a longer-horizon consistency, we consider all frame pairs from the entire length *T*.

Additionally, we compare three metrics as done by recent work [20, 26, 27]. ${\mathcal {M}}_{tOF}$ measures the differences in optical flow fields between original and translated sequences [27]. ${\mathcal {M}}_{tLP}$ compares feature distances across sequences [26]. Finally, ${\mathcal {M}}_{W}$ measures warping errors using flow from the source sequence in the target domain. Visualizations for and detailed definitions of these metrics can be found in Chapter 4 of the supplementary material.

**Evaluating image-translation quality** We assess the visual quality of individual generated samples by comparing the translations of the test splits with the target domain samples. For comparison, we use the FID and KID metrics [20] and the LPIPS diversity [26]. The FID and KID metrics empirically estimate the distributions of both sets of images and compute the divergence between them. For a translation of high quality, the generated images are expected to have a low distance to the target domain images.

**Evaluating downstream task performance** Ideally, translating data from one domain into another makes it directly usable for downstream applications. To evaluate this desired property, we conduct two experiments: First, we evaluate a phase-classification model, described in Chapter 7 of the supplementary, on translated data. The model is pre-trained on the training split of Cataract101 and evaluated on the translations of the CATARACTS test split coming from each approach above. These evaluations reveal how many of the relevant perceptual properties are successfully translated into correct properties of the target domain.

Second, we retrain the phase classification model on a combined dataset. This dataset consists of the Cataract101 training data and the *MT-UNIT* translations of the full CATARACTS dataset into the Cataract101 domain. The performance is compared against training the model solely on the Cataract101 training data. The evaluations will show whether domain transfer models can successfully be used to overcome class imbalances, if more of the relevant classes are available in the other domain. For an overall schematic depiction of our evaluation procedure, see Chapter 5 of the supplementary material.

## Results

Table 1 displays the results for evaluating the temporal consistency of translated sequences. Our proposed method almost always outperforms the baselines concerning the ${\mathcal {M}}^\textrm{SSIM}_{TC}$, ${\mathcal {M}}_{tOF}$, ${\mathcal {M}}_{tLP}$ and ${\mathcal {M}}_{W}$ metrics. In contrast, the CycleGAN-based methods dominate the ${\mathcal {M}}^\textrm{RMSE}_{TC}$ metric, which we attribute to these approaches’ high number of static predictions and mode-collapses. As a result, the foreground masks show lower values. Those will not have a high difference from sequences with very infrequent movements, which is often the case for our data. The spatial metrics ${\mathcal {M}}^\textrm{RMSE}_{TC}$ and ${\mathcal {M}}_{W}$ need to reflect our qualitative findings better. They should only be evaluated together with other quantities since simple solutions, like blurry translations, easily achieve good values for them [20, 27]. In Chapter 6 of the supplementary, we show ablation studies for both main components of our approach.

**Table 1:** Quantitative inter-sequence temporal consistency evaluation

	${\mathcal {M}}^\textrm{SSIM}_{TC}$ $(\uparrow )$	${\mathcal {M}}^\textrm{RMSE}_{TC}$ $(\downarrow )$	${\mathcal {M}}_{tOF}$ $(\downarrow )$	${\mathcal {M}}_{tLP}$ $(\downarrow )$	${\mathcal {M}}_{W}$ $(\downarrow )$
CATARACTS $\rightarrow$ Cataract101					
CycleGAN	11.4290	0.2042	1.6001	0.0401	0.1731
RecycleGAN	11.6594	**0**.**1908**	1.4201	0.0308	0.1665
UNIT	10.1807	0.2694	1.7202	0.0525	0.1848
OF-UNIT	10.4387	0.2583	1.5722	0.0496	0.1804
MT-UNIT (OURS)	**11**.**9856**	0.2163	**1**.**2579**	**0**.**0304**	**0**.**1648**
Cataract101 $\rightarrow$ CATARACTS					
CycleGAN	13.9807	**0**.**1057**	0.8325	0.0349	**0**.**0776**
RecycleGAN	13.9525	0.1116	0.8004	0.0298	0.1414
UNIT	11.6594	0.1667	1.7219	0.0534	0.1061
OF-UNIT	11.1979	0.1749	1.7343	0.052	0.1069
MT-UNIT (OURS)	**14**.**9375**	0.155	**0**.**5825**	**0**.**0215**	0.0985

The bold values mark the best results for the corresponding metric. If mean and std are displayed, then the best mean value is marked bold

**Fig. 4:**
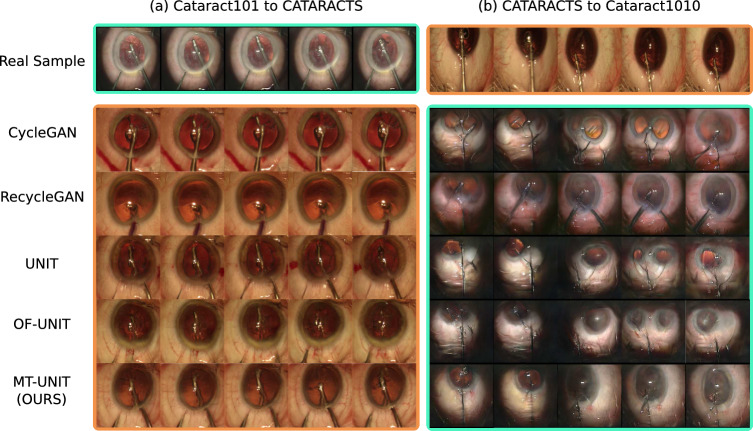
Qualitative results from translating a Cataract101 sequence (turquoise) into the CATARACTS domain (orange) and vice-versa. For the displayed sequences, our method shows increased temporal consistency and tool preservation

**Image-translation quality** Table 2 displays the frame-wise image quality evaluations. While CycleGAN achieves the lowest FID and second lowest KID scores for the translation from CATARACTS to Cataract101, the performance of the CycleGAN-based methods decreases rapidly in the other direction due to many failure cases and mode collapses. This decrease in performance is well reflected when evaluating the image diversity by computing the average LPIPS feature distance between randomly sampled pairs of images of each translated dataset. On the contrary, our approach consistently scores at least second in both directions. Figure 4 displays qualitative examples, showing many more inconsistencies and failure modes for the baseline methods.

**Table 2:** Quantitative image translation evaluation

Method	CATARACTS $\rightarrow$ Cataract101			Cataract101 $\rightarrow$ CATARACTS		
	FID $(\downarrow )$	KID $(\downarrow )$	Diversity $(\uparrow )$	FID $(\downarrow )$	KID $(\downarrow )$	Diversity $(\uparrow )$
CycleGAN	**112**.**8**	.0978 ±.0096	**.2794** ± **.002**	287.2	.3929 ±.0375	.1084 ±.0083
RecycleGAN	125.9	.1188 ±.0121	.2578 ±.0016	278.1	.3486 ±.039	.0707 ±.0111
UNIT	128.2	.1216 ±.012	.2712 ±.0019	129.8	.1209 ±.0122	.1752 ±.0045
OF-UNIT	121.5	.1091 ±.0104	.2721 ±.0018	**116**.**3**	**.1058** ± **.0118**	.1829 ±.0038
MT-UNIT (OURS)	115.3	**.0919** ± **.0092**	.2787 ±.0018	116.9	.1131 ±.0139	**.2148** ± **.0029**

The bold values mark the best results for the corresponding metric. If mean and std are displayed, then the best mean value is marked bold

**Downstream task performance** We report the downstream task experiments’ F1, AUROC and average precision (AP) scores in Table 3. Chapter 8 of the supplementary material gives an overview of the performance per class and a visualization of a sequence sample. To evaluate the significance of our *normal* vs *extended* experiments, we conduct a student’s *t*-test for the hypothesis of an increased F1 score when training on *extended* data. The test is conducted on $N=30$ splits of the data and tested against a $p=0.005$ significance level. The results reveal significant improvements with a test statistic of $t=3.0017$ using the *extended* data. We find that imposing spatio-temporal constraints increases the usability of the generated data for downstream tasks, and generating synthetic data can overcome class imbalances.

**Table 3:** Performance evaluation for the downstream task of surgical phase classification

Method	CycleGAN	UNIT	RecycleGAN	OF-UNIT	MT-UNIT (OURS)	*Normal*	*Extended*
F1 score	.171 ± .015	.223 ± .015	.201 ± .016	.285 ± .02	**.296 ± .024**	.767 ± .036	**.786 ± .027**
AUROC	.312 ± .083	.356 ± .108	.328 ± .092	.356 ± .108	**.378 ± .121**	.936 ± .001	**.945 ± .001**
AP	.224 ± .021	.19 ± .017	.223 ± .017	.265 ± .027	**.297 ± .038**	.615 ± .038	**.626 ± .044**

The bold values mark the best results for the corresponding metric. If mean and std are displayed, then the best mean value is marked bold

## Conclusion

Transitioning from Im2Im to Seq2Seq translation requires additional spatio-temporal constraints to produce realistic sequences. Therefore, we propose several methodologies to evaluate the temporal consistency of translated sequences, and we present a novel method to improve in that regard. Our approach can produce more realistic sequences based on the translation of movement between domains. Our evaluations have shown improved consistency in the CATARACTS and Cataract101 domains. Baseline methods often induce a bias to consistent samples, risking the reduction of Im2Im quality. We show our method stays competitive regarding per-frame translation quality.

Nevertheless, many failure cases remain, which are expound in Chapter 9 of the supplementary. We will explore ways to improve upon our approach by imposing a tighter structure into the shared latent space across domains. We have shown that incorporating temporal information into domain transfer methods yields significant improvements when striving for data that downstream applications require. This eliminates the intrinsic limitations that surgical datasets contain, e.g., by smoothing out label distributions and ensuring realistic sequential images. By improving the performance of clinical applications with synthetic data, we move closer toward efficient computer assisted treatments.

## Supplementary information

The supplementary material includes details about the full pipeline, training and evaluation schemes, as well as further results of our ablation- and downstream task studies. Links to the datasets are provided here.

## Supplementary Information

Below is the link to the electronic supplementary material.

Supplementary file 1 (pdf 1692 KB)

## Data Availability

Code is available at https://github.com/MECLabTUDA/TC-Seq2Seq.
