# Supplementary material for: Temporally consistent sequence-to-sequence translation of cataract surgeries
Source: Int J Comput Assist Radiol Surg. 2023 May 23;18(7):1217–24. doi: 10.1007/s11548-023-02925-y (PMC10329626; doi:10.1007/s11548-023-02925-y)
Supplement: Supplementary file 1 — (pdf 1692 KB) [file 11548_2023_2925_MOESM1_ESM.pdf]

Temporally Consistent Sequence-to-Sequence Translation of Cataract Surgeries - Supplementary Information

Yannik Frisch[1*], Moritz Fuchs[1] and Anirban Mukhopadhyay[1]

[1]GRIS, TU Darmstadt, Fraunhoferstraße 5, Darmstadt, 64283, Hessen, Germany.

*Corresponding author(s). E-mail(s): yannik.frisch@gris.tu-darmstadt.com;
Contributing authors: moritz.fuchs@gris.tu-darmstadt.com; anirban.mukhopadhyay@gris.tu-darmstadt.com;

1 UNIT (VAE-GAN) Training Objectives

For a given sampled sequence $a \in A$ consisting of two consecutive frames, we encode it into the latent space which is shared across encoders and decoders, denoted as $z_A := E_A(a)$. From the latent representation, the model should reconstruct the sample when forwarding it through G_A, which the VAE objective enforces:

$$\mathcal{L}_{VAE_A}(E_A, G_A) = \lambda_1 KL(q_A(z_A|a)||p_\eta(z)) - \lambda_2 \mathbb{E}_{z_A \sim q_A(z_A|a)}[\log p_{G_A}(a|z_A)] \tag{1}$$

where $p_\eta(z) := \mathcal{N}(z|0, I)$. The translation into domain B is trained using the GAN objective:

$$\mathcal{L}_{GAN_A}(E_A, G_A, D_A) = \lambda_0 \mathbb{E}_{a \sim A}[\log D_A(a)] + \lambda_0 \mathbb{E}_{b \sim q_B(z_B|b)}[\log(1 - D_A(G_A(z_B))] \tag{2}$$

A VAE-like objective also models the cycle-consistency constraint:

$$\mathcal{L}_{CC_A}(E_A, G_A, E_B, G_B) = \lambda_3 KL(q_A(z_A|a)||p_\eta(z)) + \lambda_3 KL(q_B(z_B|\hat{b})||p_\eta(z)) - \lambda_4 \mathbb{E}_{z_B \sim q_B(z_B|\hat{b})}[\log p_{G_A}(a|z_B)] \tag{3}$$

We further employ a commonly used perceptual loss between a sample a and its translation $\hat{b}$ by computing the distance between features of both images, obtained by a pre-trained perceptual VGG network V:

$$\mathcal{L}_{VGG_A} = \lambda_5 ||V(a) - V(\hat{b})||_2^2 \tag{4}$$

The sum of the terms $\mathcal{L}_{VAE}$, $\mathcal{L}_{GAN}$, $\mathcal{L}_{CC}$ and $\mathcal{L}_{VGG}$ gives the overall objective for both domains.

2 Flow-Based Image Warping

The *Optical Flow* (OF) F_A between two consecutive frames a_t and a_{t+1} of domain A represents their pixel-wise displacement over time.

If we have estimated such a flow-field F_A (e.g. using the Gunnar Farneback algorithm or a pre-trained RAFT model [1]), we can recreate a_t from a_{t+1} and vice-versa [2, 3].

Springer Nature 2021 LaTeX template

3 Architecture Depiction

Figure 1 displays our entire generative pipeline, omitting the discriminators. Given two consecutive frames a_t and a_{t+1} from source domain A, the VAE-GAN backbone translates them into $\hat{b}_t$ and $\hat{b}_{t+1}$. The adversarial objective in equation 2 constrains translated frames to resemble samples from domain B. A pre-trained RAFT model [1] extracts the optical flow F_A between the source domain frames. Our motion translation module then translates F_A into $\hat{F}_B$ depending on both source frames and the previous translated target frame $\hat{b}_t$. The translated flow is used to warp this target frame into $\tilde{f}(\hat{b}_t, \hat{F}_B)$. Eventually a motion translation loss between $\hat{b}_t$ and $\tilde{f}(\hat{b}_t, \hat{F}_B)$ constrains the motion translation module to produce realistic motion for domain B. Furthermore, a time-invariant MS-SSIM loss is imposed over consecutive frames $\hat{b}_t$ and $\hat{b}_{t+1}$, to strengthen the sequential generation of frames.

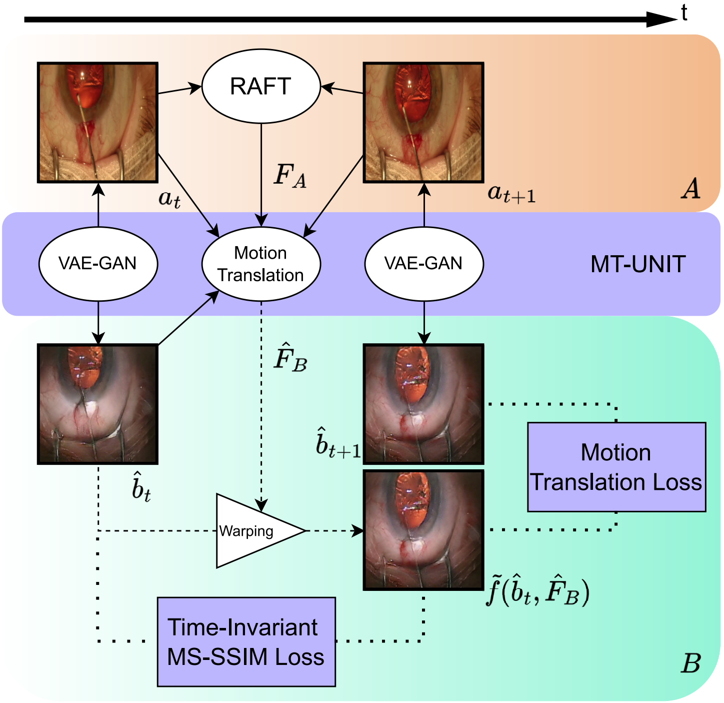

Fig. 1 Generative pipeline. This diagram depicts the full generative pipeline of our proposed Seq2Seq translation model. Given consecutive frames a_t and a_{t+1} of the source domain A, we predict the optical flow F_A between them using a pre-trained *RAFT* model [1]. The Motion Translation module is trained to translate this flow into a flow field $\hat{F}_B$ that produces consistent movements for the translated sequence in the target domain B.

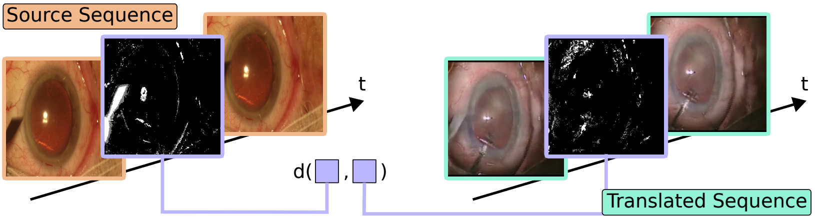

Fig. 2 Consistent translations should have similar differences between frames. One quantity of interest here are the differences in foreground masks extracted from consecutive frames (purple). Besides a scaling factor these masks should be similar. A big difference indicates a vastly different movement in the source sequence (orange) and the translated sequence (turquoise). Small local differences indicates a non-smooth translation of textures.

4 Temporal Consistency Metrics

To evaluate the temporal consistency of translated image sequences, we propose to compare the differences in extracted foreground masks fg of a sequence $a \in A$ and its translation $\hat{b}$ by

$$\mathcal{M}_{TC}^{d} = \frac{1}{T(T-1)} \sum_{t'=1}^{T} \sum_{t=1}^{T} d(|fg(a_t) - fg(a_{t'})|, |fg(\hat{b}_t) - fg(\hat{b}_{t'})|) \tag{5}$$

where d is a distance function, as visualized in Figure 2. In our experiments, the foreground extraction is realized using *MOG2* [4]. For possible choices of d, we explore the Root Mean Squared Error (RMSE) and the Structural Similarity Index Metric (SSIM):

For an optimal consistent translation of sequences we would like to achieve the same smooth transitions in the translated sequence as in the input sequence. This includes the overall global movement that is happening between the frames. E.g. if the pupil is moving from left to right in the source sequence, we do not want the pupil to stand still in the translated sequence, as it would happen with a mode collapse of the generator. Though, smooth transitions would also include smooth displacements of the displayed textures: If the eyeball is not moving in the source frame, we do not want the vessels on the sclera to change.

The latter displacements will produce tiny patches of high values in the corresponding foreground masks. When we compare these masks with the source masks, then using the RMSE will yield high metric values. The closer the $\mathcal{M}_{TC}^{RMSE}$ gets to zero, the better the alignment of the translated textures and movements. Though, there might be sequences where equal entities have different textures or entities have to be scaled across domains. The corresponding foreground masks show different activations and will result in higher metric values.

As an alternative, we can use a perceptual metric like the SSIM directly on both foreground masks. This metric is less prone to small local differences and scaling. Therefore, higher values of $\mathcal{M}_{TC}^{SSIM}$ indicate that the global movement

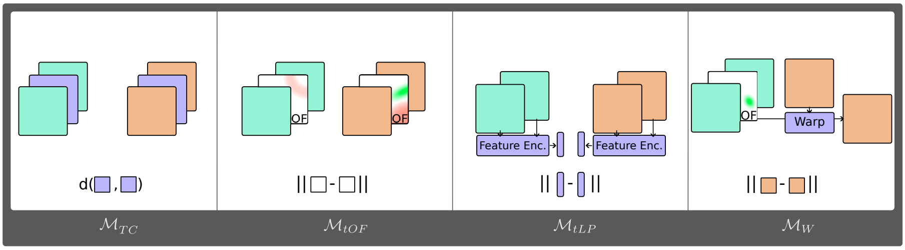

Fig. 3 Illustrations of all metrics used during evaluation. $\mathcal{M}_{TC}$ is comparing residuals between images. $\mathcal{M}_{tOF}$ is computing the distance of optical flow fields between sequences. For $\mathcal{M}_{tLP}$ a feature encoder extracts feature representations, whose differences are compared across domains. Finally, $\mathcal{M}_{W}$ uses the extracted flow of the source sequence the evaluate if it can be used to warp frames of the target sequence.

of entities is not correctly translated, e.g. when a tool is moving smoothly in the input sequence but disappears in some frames of the translated sequence (see for example Figure 2. On the contrary, values closer to 0 indicate that the global movement of entities in the scene is better translated. Additionally, we make use of the following metrics, as proposed in recent work [5–7]:

First, we compare the optical flow between consecutive frames of the translated sequence and the original sequence, which computes as

$$\mathcal{M}_{tOF} = \frac{1}{T}\sum_{t=2}^{T} ||W(a_t, a_{t-1}) - W(\hat{b}_t, \hat{b}_{t-1})||_1 \tag{6}$$

where W is a pre-trained optical-flow estimator. In our experiments we use the *RAFT* model [1] for W. Higher values stem from a discrepancy in the optical flow between the sequences. This metric is not robust against scaling (using the non-translated flow) and textural differences of equal entities across domains.

Second, we compare the frames' perceptual feature distances

$$\mathcal{M}_{tLP} = \frac{1}{T}\sum_{t=2}^{T} ||LP(a_t, a_{t-1}) - LP(\hat{b}_t, \hat{b}_{t-1})||_1 \tag{7}$$

using the perceptual LPIPS metric. Values close to 0 indicate that the perceptual differences between consecutive frame-pairs of both sequences is similar. This metric heavily depends on the performance of a pre-trained feature extractor. To correctly penalize only non-smooth transitions, the extractor has to map semantically similar frames (and therefore their distances) to similar feature vectors.

Finally, we examine the warping error

$$\mathcal{M}_{W} = \frac{1}{T}\sum_{t=1}^{T} ||\hat{b}_t - \tilde{f}(\hat{b}_{t-1}, (W(a_t, a_{t-1}))||_2^2 \tag{8}$$

where $\tilde{f}$ is the warping operation, using the estimated flow from the source domain to warp the frame in the target domain sequence. This metric evaluates if the source domain flow (translated or not) can be used to successfully warp the translated frames. The lower the metric values are, the more the translated sequences and the (translated) flow align spatially. All metrics are visualized schematically in Figure 3.

5 Evaluation Procedure

Our evaluation procedure is laid out schematically in Figure 4.

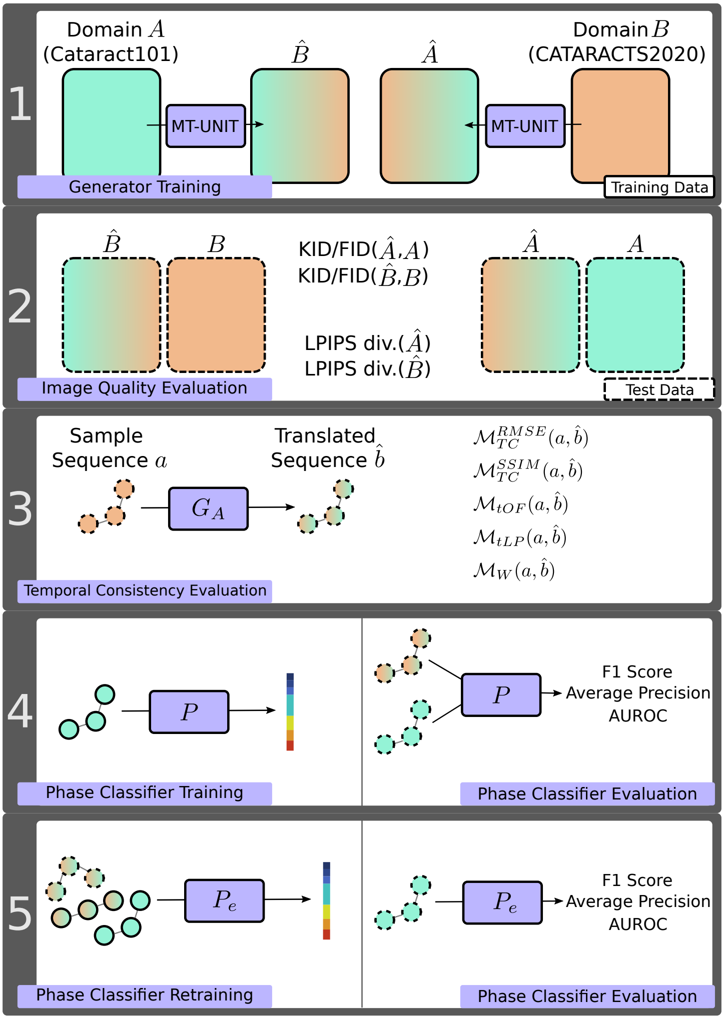

Fig. 4 Full evaluation procedure. We first train our MT-UNIT model to translate frames between domains A and B **(1)**. Then we evaluate the model regarding the image quality **(2)** and temporal consistency **(3)** of translated test sequences. Following that, we train a phase classifier on domain A and evaluate it on the domain's test samples and translated test samples from domain B **(4)**. Subsequently, we retrain the phase classifier after extending the training set from domain A with translated samples from both - the test and the training set of domain B - and finally evaluate it once more on the test set of domain A **(5)**.

6 Ablation Study: Importance of Motion Translation and Time-Invariant Structural Similarity

We carried out ablation studies to highlight the importance of both components of our proposed method. We evaluate the performance of our model without the motion translation module (*w/o MT*) and without the time-invariant structural-similarity loss (*w/o SSIM*). Table 1 displays the results. Note that the model without the motion translation module achieved a constantly lower score for $\mathcal{M}_{tOF}$, which is expected since the motion translation module adapts the target sequence flow based on the spatial properties of both domains. This adapted flow is significantly different from the source sequence flow if both domains differ greatly in their spatial arrangements, which is often the case between CATARACTS and Cataract101. Overall we found that both components yield a performance increase.

Table 1 Ablation study results.

	**CATARACTS → Cataract101**				
**Metric**	$\mathcal{M}_{TC}^{SSIM}$ (↑)	$\mathcal{M}_{TC}^{RMSE}$ (↓)	$\mathcal{M}_{tOF}$ (↓)	$\mathcal{M}_{tLP}$ (↓)	$\mathcal{M}_{W}$ (↓)
UNIT	10.1807	0.2694	1.7202	0.0525	0.1848
w/o MT	10.9073	0.2317	**1.1375**	0.0355	0.184
w/o SSIM	11.0199	0.2176	1.2905	0.0451	0.1756
MT-UNIT	**11.9856**	**0.2163**	1.2579	**0.0304**	**0.1648**
	**Cataract101 → CATARACTS**				
**Metric**	$\mathcal{M}_{TC}^{SSIM}$ (↑)	$\mathcal{M}_{Tc}^{RMSE}$ (↓)	$\mathcal{M}_{tOF}$ (↓)	$\mathcal{M}_{tLP}$ (↓)	$\mathcal{M}_{W}$ (↓)
UNIT	11.6594	0.1667	1.7219	0.0534	0.1061
w/o MT	13.6513	0.14	**0.4908**	0.04	0.1069
w/o SSIM	11.3445	0.1522	1.5403	0.0522	0.1203
MT-UNIT	**14.9375**	**0.155**	0.5825	**0.0215**	**0.0985**

7 Phase classification model

The downstream task model architecture is explained in Table 2. To incorporate temporal information, we concatenate three sequential frames to form a nine-channel tensor that yields as the input to our model. Every intermediate convolutional layer is followed by a LeakyReLU nonlinearity with a negative slope of 0.2. Additionally, we use BatchNorm2d layers after Conv2, Conv3 and Conv4. The final convolutional layer maps to the desired number of classes, $k = 11$ in the case of Cataract-101.

Table 2 Downstream task model.

Layer	Input Ch.	Conv. Kernel	Stride	Padding	Output Ch.
Conv1	9	4	7	1	64
Conv2	64	4	2	1	128
Conv3	128	4	2	1	256
Conv4	256	4	1	1	512
Conv5	512	4	1	1	k

8 Downstream Task Phase-Wise Performance

Figure 5 shows the per-phase F1 score for the two different experiments of our last downstream task evaluation.

First (*normal*, orange), we train a multi-frame phase classification model on the training dataset of Cataract101 and evaluate it on the test split of this dataset.

Next (*extended*, blue), we artificially increase the amount of training data by translating the CATARACTS training and test data (pre-filtered for matching phases). Then we retrain the model and again evaluate it on the Cataract101 test split to see if the artificial data can help with the performance for underrepresented classes.

The results show increased scores for the labels that yield more samples after the extension. Note that the performance for untranslated phases slightly decreases, which explains the only marginal increase in the overall F1 score from 0.767 to 0.786. This problem is very closely related to the problem of forgetting in continual learning, and we plan to address it in future research. A classifciation example for one sequence is displayed in Figure 6.

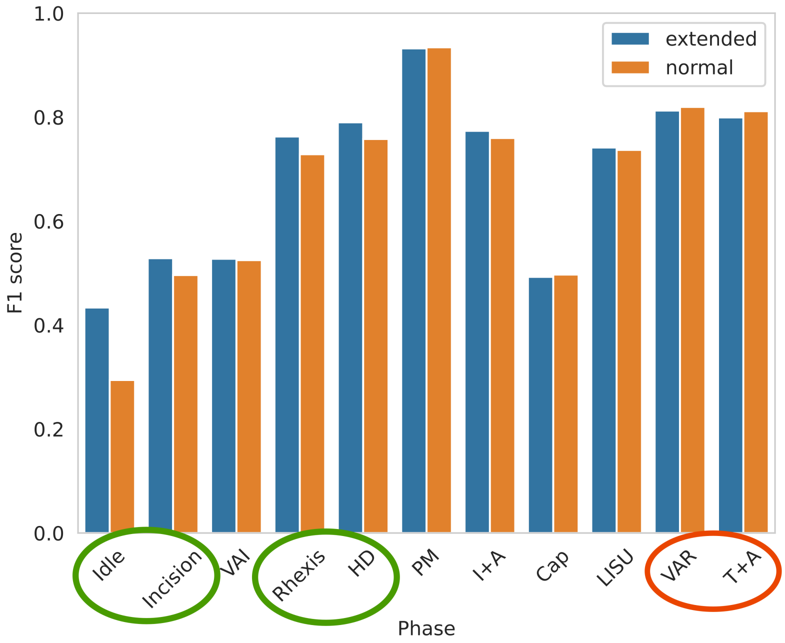

Fig. 5 Label-wise F1 score of phase-classification model. The graph compares the performance when training on the original Cataract101 training data (orange) vs training on the artificially extended training data (blue). Note how classes that have been artificially increased (green) yield increased performance, while not translated classes only drop slightly (red).

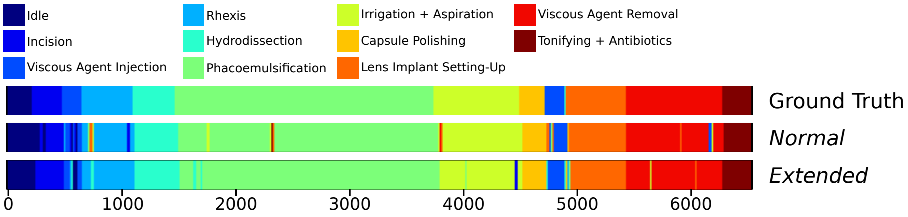

Fig. 6 Phase segmentations of Cataract101 video *846*. Training the phase predictor on the artificially *extended* data improves the prediction of surgical phases.

9 Limitations and Failure Cases

The proposed temporal constraints increase the usability of translated data for downstream applications. Nonetheless, the first part of our downstream evaluations still reveals room for improvement. While achieving higher temporal consistency, complete tool preservation compared to the source sequence remains a problem, as shown in Figure 7. The evaluated approaches will often collapse onto always displaying a specific tool in the output sequences or not showing any tool. We think that further constraints on the latent space could improve upon this issue, e.g. in the form of weak supervision or structured representations.

We found another limitation in the applicability for cases without direct matches in the target domain, e.g. if certain tools from CATARACTS are not present in the Cataract101 dataset. Alternatively, when Cataract101 samples show zoom levels that are not present in the CATARACT data. We contribute the latter to the performance decrease when translating from Cataract101 to CATARACTS, compared to the other way round. The approaches assume a certain degree of perceptual similarities in the domains. When, for example, a plastic container that contains the artificial lens is held in front of the camera, this is not the case. Further guiding the latent space will also improve upon this problem, and it is one part we will address in future research.

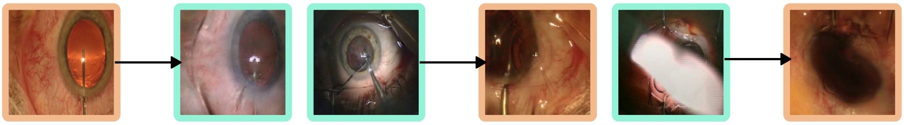

Fig. 7 Typical failure cases. Lacking tool preservation (left), zoom level discrepancies (middle) and objects in front of the camera that occlude the eye (right).

10 Data-Sets

We use publicly available datasets in our experiments:

- CATARACTS2020
- Cataract101

11 Video Examples of Translated Sequences

Video files of original and translated sequences are provided at https://hessenbox.tu-darmstadt.de/getlink/fiXb3gSKVNPvtuwq54rNEbvN/.

References

[1] Teed, Z., Deng, J.: Raft: Recurrent all-pairs field transforms for optical flow. In: ECCV, pp. 402–419 (2020). Springer

[2] Farnebäck, G.: Two-frame motion estimation based on polynomial expansion. In: Scandinavian Conference on Image Analysis, pp. 363–370 (2003). Springer

[3] Bouguet, J.-Y., *et al.*: Pyramidal implementation of the affine lucas kanade feature tracker description of the algorithm. Intel corporation **5**(1-10), 4 (2001)

[4] Zivkovic, Z.: Improved adaptive gaussian mixture model for background subtraction. In: Proceedings of the 17th International Conference on Pattern Recognition, 2004. ICPR 2004., vol. 2, pp. 28–31 (2004). IEEE

[5] Chu, M., Xie, Y., Mayer, J., Leal-Taixé, L., Thuerey, N.: Learning temporal coherence via self-supervision for gan-based video generation. ACMTOG **39**(4), 75–1 (2020)

[6] Rivoir, D., Pfeiffer, M., Docea, R., Kolbinger, F., Riediger, C., Weitz, J., Speidel, S.: Long-term temporally consistent unpaired video translation from simulated surgical 3d data. In: ICCV, pp. 3343–3353 (2021)

[7] Zhang, R., Isola, P., Efros, A.A., Shechtman, E., Wang, O.: The unreasonable effectiveness of deep features as a perceptual metric. In: CVPR, pp. 586–595 (2018)
